# 热消融治疗原发性和转移性肺部肿瘤专家共识（2017年版）

**DOI:** 10.3779/j.issn.1009-3419.2017.07.01

**Published:** 2017-07-20

**Authors:** 欣 叶, 卫君 范, 徽 王, 俊杰 王, 善智 古, 威健 冯, 一平 庄, 宝东 刘, 晓光 李, 玉亮 李, 坡 杨, 霞 杨, 武威 杨, 俊辉 陈, 嵘 张, 征宇 林, 志强 孟, 凯文 胡, 晨 柳, 忠民 彭, 玥 韩, 勇 靳, 光焰 雷, 博 翟, 广慧 黄, 中国抗癌协会肿瘤微创治疗专业委员会肺癌微创治疗分会

**Affiliations:** 1 250014 济南, 山东大学附属省立医院肿瘤科 Department of Oncology, Shandong Provincial Hospital Affiliated to Shandong University, Ji'nan 250014, China; 2 510060 广州, 中山大学肿瘤医院影像与微创介入中心 Imaging and Interventional Center, Sun Yat-sen University Cancer Center, Guangzhou 510060, China; 3 130012 长春, 吉林省肿瘤医院介入治疗中心 Interventional Treatment Center, Jilin Provincial Tumor Hospital, Changchun 130012, China; 4 100191 北京, 北京大学第三医院放射治疗科 Department of Radiation Oncology, Peking University 3rd Hospital, Beijing 100191, China; 5 410013 长沙, 湖南省肿瘤医院放射介入科 Department of Interventional Therapy, Hunan Provincial Tumor Hospital, Changsha 410013, China; 6 100045 北京, 首都医科大学附属复兴医院肿瘤科 Department of Oncology, Fuxing Hospital Affiliated to the Capital University of Medical Sciences, Beijing 100045, China; 7 210009 南京, 江苏省肿瘤医院介入科 Department of Interventional Therapy, Jiangsu Cancer Hospital, Nanjing 210009, China; 8 100053 北京, 首都医科大学宣武医院胸外科 Department of Thoracic Surgery, Xuanwu Hospital Affiliated to the Capital University of Medical Sciences, Beijing 100053, China; 9 100005 北京, 北京医院肿瘤微创中心 Department of Tumor Minimally Invasive Therapy, Beijing Hospital, Beijing 100005, China; 10 250033 济南, 山东大学第二医院介入治疗中心 Interventional Treatment Center, Shandong University Second Hospital, Ji'nan 250033, China; 11 150001 哈尔滨, 哈尔滨医科大学第四人民医院介入放射科 Department of Interventional Radiology, The Fourth Hospital of Harbin Medical University, Harbin 150001, China; 12 100071 北京, 解放军307医院肿瘤微创治疗科 Department of Tumor Minimally Invasive Therapy, 307 Hospital, Beijing 100071, China; 13 518036 深圳, 北京大学深圳医院微创介入科 Department of Minimally Invasive Interventional Therapy, Shenzhen Hospital of Beijing University, Shenzhen 518036, China; 14 350005 福州, 福建医科大学附属第一医院介入科 Department of Interventional Therapy, the First Affiliated Hospital of Fujian Medical University, Fuzhou 350005, China; 15 200032 上海, 复旦大学肿瘤医院微创治疗科 Department of Integrative Oncology, Fudan University Shanghai Cancer Center, Shanghai 200032, China; 16 100078 北京, 北京中医药大学东方医院肿瘤科 Department of Oncology, Dongfang Hospital Affiliated to Beijing University of Chinese Medicine, Beijing 100078, China; 17 100083 北京, 北京肿瘤医院介入治疗科 Department of Interventional Therapy, Beijing Cancer Hospital, Beijing 100083, China; 18 250014 济南, 山东省立医院胸外科 Department of Thoracic Surgery, Shandong Provincial Hospital Affiliated to Shandong University, Ji'nan 250014, China; 19 100021 北京, 中国医学科学院肿瘤医院介入治疗科 Department of Interventional Therapy, Tumor Institute and Hospital, Chinese Academy of Medical Sciences, Beijing 100021, China; 20 215004 苏州, 苏州大学第二附属医院介入治疗科 Department of Interventional Therapy, the Second Affiliated Hospital of Soochow University, Suzhou 215004, China; 21 710061 西安, 陕西省肿瘤医院胸外科 Department of Thoracic Surgery, Shanxi Provincial Tumor Hospital, Xi'an 710061, China; 22 200127 上海, 上海交通大学仁济医院肿瘤介入治疗科 Tumor Interventional Therapy Center, Shanghai Renji Hospital, Shanghai 200127, China

## 1 前言

在世界范围内肺癌居癌症发病率和死因之首，全球每年发病约250万人，每年有超过160万人死于肺癌^[1]^。在我国肺癌的发病形势更加严峻，2010年新发肺癌605, 900人，死亡486, 600人^[2]^。估计2015年我国将新发肺癌733, 300人，死亡610, 200人^[3]^，绝对数均排在世界第一。对于早期非小细胞肺癌（non-small cell lung cancer, NSCLC），外科切除是治愈的主要手段^[4]^，但是由于各种原因，大约80%的肺癌无法通过手术切除治疗。对于无法手术切除的多数肺癌患者在传统的放化疗中获益有限，因此许多新的局部治疗方法应运而生，包括局部消融治疗等。局部热消融术作为一种微创技术已经应用于早期肺癌的治疗，每年治疗肺癌患者的例数迅速增加^[5-16]^。肺部转移瘤在临床上十分常见，是所有肿瘤转移的第二器官，目前已证实经皮热消融也可以有效地治疗肺部转移瘤^[17-20]^。2014年3月1日国内有关肿瘤微创诊治领域的18位知名专家在济南召开会议，共同讨论达成了“热消融治疗原发性和转移性肺部肿瘤的专家共识（2014年版）”，以下简称为“共识（2014）”，并发表在《中国肺癌杂志》上^[21]^，2015年英文版在《*Thoracic Cancer*》杂志上发表^[22]^。“共识（2014）”发表 3年多来，其为我国热消融治疗肺部肿瘤的发展起到了积极促进作用，同时也为国际热消融治疗肺部肿瘤的发展做出了贡献。但是在3年多的临床实践中我们也发现“共识（2014）”有许多不足之处，需要进一步完善。为了融合国内外先进的“精准医学”概念和适应肿瘤消融技术突飞猛进的发展，为了推动我国临床肿瘤微创技术的发展和提高肺部恶性肿瘤多学科综合治疗的水平，为了加强国内各医院肺癌微创诊疗专业之间的交流和更好地规范热消融治疗肺部恶性肿瘤技术，“中国抗癌协会肿瘤微创治疗专业委员会肺癌微创治疗分会”于2017年4月16日在北京组织了包括胸部外科、放射治疗科、肿瘤内科、介入医学科、影像科、中医肿瘤科等国内多学科有关知名专家对“共识（2014）”进行修订，并通过电子邮件的形式征求了全体“中国抗癌协会肿瘤微创治疗专业委员会肺癌微创治疗分会”委员的意见，其目的是能更好地为临床实践和规范发展热消融治疗肺部肿瘤提供参考。

## 2 肿瘤热消融的概念

随着不可逆电穿孔消融技术（irreversible elect-roporation）^[23-26]^的出现，肿瘤消融的概念发生了较大的变化，热消融（thermal ablation）属于能量消融（energy-based ablation）^[27]^的一种。肿瘤热消融是针对某一脏器中特定的一个或多个肿瘤病灶，利用热产生的生物学效应直接导致病灶组织中的肿瘤细胞发生不可逆损伤（irreversible injury）或凝固性坏死（coagulation necrosis）的一种精准微创治疗技术。在我国属于限制性医疗技术[《限制临床应用的医疗技术（2015版）》]。

## 3 局部热消融技术

热消融治疗技术，目前主要包括射频消融（radiofrequency ablation, RFA）、微波消融（microwave ablation, MWA）、冷冻消融（cryoablation）、激光消融（laser ablation）和高强度聚焦超声（high-intensity focused ultrasound, HIFU）消融^[27, 28]^，HIFU消融很少用于肺部肿瘤的消融治疗。

### 3.1 RFA

RFA是目前治疗实体瘤应用最广泛的消融技术，其原理是将射频电极穿刺入肿瘤组织中，在375 kHz-500 kHz的高频交变电流作用下，肿瘤组织内的离子相互磨擦、碰撞而产生热生物学效应，局部温度可达60 ℃-120 ℃，当组织被加热至60 ℃以上时，可引起细胞凝固性坏死。RFA消融体积取决于局部射频消融产生的热量传导与循环血液及细胞外液间的热对流^[27-29]^。2007年12月美国食品药品监督局（Food and Drug Administration, FDA）批准了RFA可以用于肺部肿瘤的治疗^[30]^，2009年以来NSCLC美国国立综合癌症网络（National Comprehensive Cancer Network, NCCN）指南、中国《原发性肺癌诊疗规范（2011年版）》（卫办医政发[2011]22号）、中国《原发性肺癌诊疗规范（2015年版）》^[31]^均推荐RFA可以用于早期不能耐受手术切除肺癌患者的治疗。

### 3.2 MWA

MWA一般采用915 MHz或2, 450 MHz两种频率。在微波电磁场的作用下，肿瘤组织内的水分子、蛋白质分子等极性分子产生极高速振动，造成分子之间的相互碰撞、相互摩擦，在短时间内产生高达60 ℃-150 ℃的高温，从而导致细胞凝固性坏死^[27, 32-34]^。由于辐射器将微波能集中在一定范围内，故而能有效地辐射到所需靶区，微波热辐射在肺内有更高的对流性和更低的热沉降效应^[35-38]^。

### 3.3 冷冻消融

氩-氦冷冻消融是目前较成熟的冷冻消融治疗技术。其原理是通过焦耳-汤姆逊（Joule-Thomson）效应，高压氩气可以使靶组织冷却至零下140 ℃，氦气可使靶组织从零下140 ℃迅速上升至零上20 ℃-40 ℃，通过这种温度梯度的变化可以导致^[39-43]^：①靶组织蛋白质变性；②细胞内外渗透压改变和“结冰”效应造成细胞裂解；③微血管栓塞引起组织缺血坏死等。用计算机断层扫描（computed tomography, CT）或磁共振成像（magnetic resonance imaging, MRI）观察到的“冰球”可以直接将消融区域与肿瘤边界进行区分，可以测定冷冻损伤的边界，这一边界大致在冰球最外缘内侧4 mm-6 mm范围内^[44]^。

上述三种消融技术是目前临床上常用的肺部肿瘤局部消融治疗技术，并各有一定优势。对于直径≤3 cm的肿瘤，三种消融方式均可获得良好的治疗效果。RFA电极的适形性好，可以通过调节消融电极来保护邻近脏器，但是受血流和气流的影响较大；对于直径＞3 cm，尤其是＞5 cm的肿瘤，MWA因其消融时间短、消融范围大，明显优于其他两种消融方式，且微波消融受到血流灌注的影响小，更加适合治疗邻近大血管的肿瘤。冷冻消融形成的“冰球”边界清晰，易于监测，可用于邻近重要脏器的肺部肿瘤。冷冻消融较少引起局部疼痛，对于肿瘤距离胸膜≤1 cm或有骨转移引起骨质破坏的肿瘤患者，冷冻消融明显优于MWA和RFA。但冷冻消融在治疗过程中消耗患者血小板，对于凝血功能差的患者，应避免使用冷冻消融。在肺部肿瘤消融中RFA在临床应用较广，积累的经验较多。由于MWA的突出优势，其在肺部肿瘤消融中应用越来越广泛^[10, 46, 47]^。

### 3.4 激光消融

肺部肿瘤的激光消融与上述三种消融比较在临床上开展相对较少，目前在激光消融中应用最广泛的是波长1, 064 nm的Nd:YAG激光（Neodymium-doped Yttrium Aluminium Garnet，钇铝石榴石晶体）^[27, 48]^。其原理为^[49]^：激光导入组织后，光子被组织生色基团所吸收后瞬间即可产生高热、压强等生物效应使肿瘤组织变性、凝固、汽化甚至炭化而达到杀灭肿瘤的目的。激光消融的特点^[50, 51]^：①消融范围较小（1.0 cm×1.5 cm），对周围组织损伤小；②由于激光能量可以瞬间释放，因此消融时间极短；③光导纤维常用21 G的Chiba针导入，因此穿刺损伤小，导致的并发症少（如出血、感染）。对于肺内多发的、重要器官旁、最大径＜1.0 cm的肿瘤有一定优势^[52, 53]^。

## 4 操作平台

### 4.1 影像引导

经皮热消融治疗的影像引导技术有^[27]^：CT、MR、超声、正电子发射型计算机断层显像-计算机断层扫描（positron emission computed tomography-CT, PET-CT）和C-臂/CT^[54-56]^等。CT是肺部肿瘤消融治疗最常用的影像引导技术，其次是MR。对于用超声能观察到肿瘤全貌的靠近胸壁或与胸壁粘连的肿瘤，可以用超声引导。C-臂/CT也有部分单位在应用。PET-CT可以进行功能成像，但临床使用较少。

### 4.2 开胸或电视胸腔镜辅助

一般用于：①肺部肿瘤邻近重要结构如大血管、肺门或心脏；②在开胸后发现肺部肿瘤不能够切除的情况下^[57]^。

## 5 肺部肿瘤热消融的适应证和禁忌证

### 5.1 治愈性消融（curative ablation）的适应证

治愈性消融是指通过热消融治疗，使局部肿瘤组织完全坏死，有可能达到治愈效果。

#### 5.1.1 原发性周围型肺癌^[5, 7, 12, 16, 58-65]^

① 患者因心肺功能差或高龄不能耐受手术切除；②拒绝行手术切除；③其他局部治疗复发后的单发病灶（如适形放疗后）；④原发性肺癌术后或放疗后肺内孤转移；⑤单肺（各种原因导致一侧肺缺如）^[66-70]^；⑥多原发肺癌，且双肺肿瘤数量≤3个。肿瘤最大径≤3 cm，且无其他部位的转移病灶。

#### 5.1.2 肺部转移瘤^[19, 20, 60, 71-75]^

某些生物学特征显示预后较好的肺内转移瘤（如肉瘤、肾癌、结直肠癌、乳腺癌、黑色素瘤和肝细胞癌）。如果原发病能够得到有效治疗，可进行肺转移瘤的消融治疗。单侧肺病灶数目≤3个（双侧肺≤5个），多发转移瘤的最大直径≤3 cm，单侧单发转移瘤的最大直径≤5 cm，且无其他部位的转移。对于双侧肺肿瘤，不建议双侧同时进行消融治疗。

### 5.2 姑息性消融（palliative ablation）的适应证

治疗的目的在于最大限度减轻肿瘤负荷、缓解肿瘤引起的症状和改善患者生活质量，对于达不到治愈性消融条件的患者，其适应证可以较治愈性消融适当放宽^[27, 60, 76-85]^。如肿瘤最大径＞5 cm或单侧肺病灶数目＞3个（双侧肺＞5个），可以进行多针、多点或多次治疗，或与其他治疗方法联合应用。如肿瘤侵犯肋骨或脊柱椎体引起的难治性疼痛，对肿瘤局部骨侵犯处进行消融，即可达到止痛效果。

### 5.3 热消融禁忌证

由于肺肿瘤患者对经皮热消融治疗具有良好的耐受性，术后肺功能几乎不受影响，因此除无法纠正的凝血障碍性疾病以外，肺部肿瘤局部热消融的绝对禁忌证相对较少^[60, 86]^。①病灶周围感染性及放射性炎症没有很好控制者，穿刺部位皮肤感染、破溃；②严重的肺纤维化，尤其是药物性肺纤维化^[86, 87]^；③有严重出血倾向、血小板小于50×10^9^/L和凝血功能严重紊乱者。抗凝治疗和/或抗血小板药物应在经皮消融前至少停用5 d-7 d；④消融病灶同侧恶性胸腔积液没有很好控制者；⑤肝、肾、心、肺、脑功能严重不全者，严重贫血、脱水及营养代谢严重紊乱，无法在短期内纠正或改善者，严重全身感染、高热（> 38.5 ℃）者；⑥有广泛肺外转移，预期生存＜3个月者^[88]^；⑦美国东部肿瘤协作组（Eastern Cooperative Oncology Group, ECOG）评分＞3分；⑧植入心脏起搏器的患者不建议使用RFA^[89-91]^。

## 6 术前准备

### 6.1 患者的评估及影像学检查

要通过认真复习病史、体格检查及近期的影像资料来评估患者的热消融适应证。适应证的选择建议多学科（胸外科、肿瘤科、放射治疗科、介入医学科、影像科等）共同讨论做出决定^[27, 60, 85, 92]^，并有消融手术前讨论记录。胸部强化CT（2周内）为消融治疗前评估的关键影像学检查，通过CT观察肿瘤的大小、位置及其与临近重要脏器、血管、气管或支气管的关系。完善相关分期检查（如骨扫描、磁共振检查），有条件者可行PET-CT检查排除或发现远处转移，对怀疑转移的纵隔淋巴结可行病理活检。对于能达到治愈性消融的患者建议消融前行PET-CT检查以便准确分期^[27, 60, 93-98]^。

### 6.2 各项实验室检查

实验室检查应包括：血常规、大小便常规、凝血功能、肝肾功能、血糖、肿瘤标记物、血型等检查，心电图、肺功能、心脏彩超（高龄患者可选）等。

### 6.3 病理检查

对于原发性肺癌，消融治疗前行经皮病灶穿刺活检或纤维支气管镜检查以明确诊断。当转移病灶不典型时建议消融治疗前对病灶进行活检^[27, 60, 99]^。

### 6.4 药品及监护设备准备

术前应准备麻醉、镇痛、镇咳、止血、扩血管、升压、降压等药物，抢救药品及设备。

### 6.5 患者准备

① 患者及/或家属（被委托人）签署知情同意书；②局部麻醉前4 h禁食，全身麻醉前12 h禁食、前4 h禁水；③手术区必要时备皮；④建立静脉通道；⑤术前口服镇咳剂；⑥患者术前教育。

## 7 麻醉与消毒

根据患者的状况，可以采用全身麻醉或局部麻醉进行消融手术^[16, 21, 100-102]^。穿刺点处用1%-2%利多卡因局部浸润麻醉，直至胸膜。对于儿童、术中不能配合、预计手术时间长、肿瘤贴近壁层胸膜可能引起剧痛的患者，建议全身麻醉。严格执行无菌操作技术规范。

## 8 消融操作

选择合适的消融技术后，CT是最常用和最准确的影像引导方式之一，操作过程就是将热消融电极（天线、探针或光纤），在CT引导下通过皮肤直接穿刺入靶组织中进行热消融。不建议在门诊进行肺部肿瘤的消融手术。

### 8.1 术前治疗计划

术前治疗计划是保证消融是否成功的关键环节主要包括：①确定肿瘤病变区域（gross tumor region, GTR）：指影像学能界定的病变区域，即确定病灶的位置、大小、形态、与邻近器官的关系，初步确定GTR；②选择合适体位及穿刺点的体表定位；③穿刺路径：指从穿刺点到达病灶的穿刺通道，此距离称为“靶皮距”；④初步制定消融参数。

### 8.2 穿刺临床靶区

麻醉后用热消融电极（天线、探针或光纤）按照术前计划的GTR，从体表定位点沿着穿刺路径逐层穿刺，穿刺深度为术前计划的“靶皮距”，然后CT扫描观察（可通过三维重建影像确认）热消融电极（天线、探针或光纤）是否到达预定的消融靶区。

### 8.3 消融靶组织

根据肿瘤的大小和部位可采用多种模式进行靶组织消融治疗：①单次单点完成消融治疗（如直径≤3 cm者）；②单次多点完成消融治疗（如直径3 cm-5 cm者）；③多电极（多天线、多探针或光纤）单次多点或多次多点完成消融治疗（如直径＞5 cm者或姑息消融）。所使用的消融参数（温度、功率、时间、循环等）根据不同的设备进行不同选择。

### 8.4 消融过程中监测

在消融过程中要用CT监测消融电极（天线、探针或光纤）是否脱靶（off target）、是否需要调整消融电极（天线、探针或光纤）、是否达到了预定消融范围、是否有术中并发症（如出血、气胸）。热消融过程中，由于热消融对肿瘤周围肺组织的损伤，在肿瘤周围可出现不透明高密度区，称为毛玻璃样影（ground-glass opacity, GGO），当GTR周围的GGO大于消融前GTR边界时，消融电极（天线、探针或光纤）可以拔出，拔出消融电极（天线、探针或光纤）时要注意消融穿刺针道。此时的靶组织定义为消融后靶区（post-ablation target zone, PTZ）。消融过程需要监测心率、血压和血氧饱和度，同时要观察患者的呼吸、疼痛、咳嗽、咯血等情况，必要时应对症处理。

### 8.5 即刻疗效评价

消融过程结束时要再次CT扫描（范围要大，最好是全肺扫描）：①初步评价操作技术的成功情况；②观察消融边界（ablative margin）：建议：如果要达到完全消融，PTZ周围的GGO至少要大于消融前GTR边界5 mm，最好达到10 mm。对于姑息消融根据临床实际情况不必达到完全消融所要求的标准，甚至不要求消融边界（如肿瘤侵犯肋骨或脊柱椎体引起的难治性疼痛）^[27, 60, 103, 104]^；③同时观察是否有并发症的发生。如果患者血压、心率及血氧饱和度正常，无咯血、气促、胸闷、呼吸困难及其他症状，可以返回病房。

### 8.6 术后处理

术后建议监测生命体征，24 h-48 h后拍胸片或CT扫描，观察是否有并发症的发生（如无症状性气胸或胸腔积液）。

### 8.7 CT引导经皮穿刺肺部肿瘤热消融操作规程

见附件。

## 9 辅助技术

消融过程中在靶组织与非靶组织之间注入液体或气体以分离靶组织与非靶组织，这样对于保护重要的非靶组织（如胸膜、心包、纵隔等）和减轻消融过程中的疼痛是十分有益的。这些技术主要包括人工液胸或人工气胸^[103, 105-109]^。

## 10 随访及疗效评估

### 10.1 随访

术后前3个月，每个月复查一次胸部增强CT。以后每3个月复查胸部增强CT或PET-CT和肿瘤标志物。主要观察局部病灶是否完全消融、肺内有无新发病灶、肺外转移以及并发症等。胸部增强CT是目前评价消融效果的标准方法，有条件的可使用PET-CT，PET-CT/强化CT两者相结合可以更准确地判断消融后的疗效。

### 10.2 术后影像学表现及疗效评估

#### 10.2.1 CT疗效评估

（1）影像学表现：热消融后由于消融区周围的出血、水肿、渗出、炎性细胞的浸润，PTZ显著大于原肿瘤的GTR，而这种影像学表现将持续3个月-6个月，因此传统的实体肿瘤疗效评价标准不适合用于热消融后局部疗效的评价^[27, 60, 110-112]^。消融后强化CT扫描显示的变化规律为：消融后1个月-3个月内病灶增大，3个月后病灶保持稳定或逐渐缩小。①早期改变（1周内）：可分为三层：a.第一层：病灶内可出现实性、蜂窝状或低密度泡影样改变（hypoattenuating bubbles）；b.第二层：围绕着消融肿瘤周边形成的GGO，一般认为GGO应超出肿瘤周边边缘至少5 mm可达到肿瘤完全消融^[27, 60, 104]^；c.第三层（外层）：在GGO外有一层密度稍高于GGO的反应带^[112]^。这种典型的影像学改变称为：“帽徽”（cockade）征象（此征象在消融后24 h-48 h更加明显）；②中期（1周-3个月内）：消融区可持续增大，GGO消失，其周边可能出现环绕清晰锐利的强化环，称为“蛋壳”（egg shell）征象^[22]^。对于靠近胸壁的肿瘤胸膜增厚也是十分常见的；③后期（3个月后）：与基线（一般以消融后4周-6周时的CT表现为基线^[27, 71, 112]^）比PTZ在3个月后病灶保持稳定，以后的CT随访过程中病灶区域有几种不同的演变模式:如缩小纤维化、空洞、结节、肺不张、消失、增大（可能复发、进展或增生纤维化）等^[113]^。（2）局部疗效评估：以消融后4周-6周时的病灶为基线判断疗效。①完全消融（出现下列表现任何一项）：病灶消失；完全形成空洞；病灶纤维化，可为疤痕；实性结节缩小或无变化或增大，但CT扫描无造影剂强化征象或/和PET-CT肿瘤无代谢活性；肺不张，肺不张内的病灶CT扫描无造影剂强化征象或/和PET-CT肿瘤无代谢活性；②不完全消融（出现下列表现任何一项）：空洞形成不全，有部分实性，且CT扫描有造影剂强化或/和PET-CT肿瘤有代谢活性；部分纤维化，病灶部分纤维化仍存有部分实性成分，且实性部分CT扫描有造影剂强化或/和PET-CT肿瘤有代谢活性；实性结节，大小无变化或增大，且伴CT扫描造影剂有强化征象或/和PET-CT肿瘤有代谢活性。

#### 10.2.2 PET-CT

PET-CT是目前判断消融后疗效最准确的手段之一，对于发现肿瘤残留、复发及远处转移是十分有益的。由于消融后的炎性反应，3个月内行PET-CT检查发现局部肿瘤残留假阳性率较高^[114]^，因此在这个阶段行PET-CT检查除能发现远处转移和新发病灶外，对于判断是否有局部残留和进展意义有限。

消融3个月后随着消融区域炎性反应的减轻或消退，PET-CT能够比较客观地反映出消融后肿瘤的代谢活性。如果PET-CT检查消融后的GTR无代谢活性，说明肿瘤达到了完全消融。如果PET-CT检查消融后的GTR有代谢活性，说明肿瘤残留或进展，未达到完全消融。在PET-CT检查中有多种模式可体现出肿瘤的代谢活性^[115]^。消融后出现肺门或纵隔淋巴结肿大是转移还是炎性反应有时十分难以确定，如果在消融后3个月肿大的淋巴结无代谢活性或代谢活性较前明显减低，则说明为炎性反应，反之则为转移。

### 10.3 临床疗效评估

在判断局部疗效的基础上，定期随访。技术成功和安全性评价至少随访6个月；初步临床疗效评价至少随访1年；中期临床疗效评价至少随访3年；长期临床疗效评价至少随访5年^[116]^。生存时间是最重要的临床疗效指标，要记录患者1年、2年、3年、5年的生存情况。对于姑息消融的患者要观察患者生存质量的改善情况（生活质量量表）、疼痛缓解情况（疼痛评分评估）、药物用量等。

## 11 并发症及处理

经皮肺肿瘤消融术是一种相对安全的局部治疗手段，其并发症的发生情况，依据美国介入放射学会（Society of Interventional Radiology, SIR）的标准^[27]^进行评估分级：①不良反应：a.疼痛；b.消融后综合征；c.无症状胸腔积液；d.影像学可见的无症状积液；e.附随的损伤；②轻微并发症：a.不需治疗，无不良后果；b.仅需简单治疗，无不良后果，包括不需要住院1天及以上的观察；③严重并发症：a.需要治疗，需要住院或住院时间延长≤48 h；b.需要重要的治疗措施，需要住院或住院时间延长＞48 h，或该并发症产生永久后遗症；c.死亡：需要说明与消融之间的关系。按照发生时间分为即刻并发症（immediate，消融后＜24 h）、围手术期并发症（periprocedural，消融后24 h-30 d）及迟发并发症（delayed，消融后＞30 d）。

### 11.1 不良反应

①疼痛：在局麻条件下手术，一般均有不同程度的疼痛（尤其是临近胸膜的病变行消融治疗时常常需要止痛治疗）。如果疼痛剧烈，可以加大阿片类止痛药物的用量，同时可以给予适量镇静剂。手术后疼痛一般为轻度疼痛，可持续数天，也有人持续1周-2周，很少出现中度以上的疼痛，可以用非甾体类药物止痛；②消融后综合征：约2/3患者可能发生，是由于坏死物质的吸收和炎性因子的释放引起。主要症状为低热、乏力、全身不适、恶心、呕吐等，一般持续3 d-5 d，少部分可能会持续2周左右。这种情况对症处理即可，必要时除给予非甾体类药物外，可以适量短时应用小剂量糖皮质激素，同时加强支持治疗；③咳嗽：消融术中出现咳嗽是十分常见的症状，剧烈的咳嗽可导致或加重气胸或皮下气肿，有时可使消融电极（天线、探针或光纤）脱靶，有时加剧患者紧张甚至不能耐受消融。引起咳嗽的原因可能与消融时局部温度增高刺激肺泡、支气管内膜或胸膜所致，术后咳嗽是肿瘤组织坏死及其周围肺组织热损伤引起的炎症反应所致。预防：术前1 h口服可待因可减轻咳嗽反应。轻度的咳嗽不影响消融手术，剧烈咳嗽要停止消融手术或间断消融。术后咳嗽可适当给予止咳化痰药以及必要的抗生素；④胸膜反应：消融过程中刺激了支配壁层胸膜的迷走神经，兴奋的迷走神经可使心率减慢、甚至心跳停止。出现这种情况要暂停消融，要充分局部麻醉，并适当应用阿托品、镇静剂等药物。

### 11.2 并发症

#### 11.2.1 气胸

气胸是消融后最常见的并发症，发生率为10%-67%^[19, 117-122]^。气胸更常见于以下情况：肺气肿、男性、年龄＞60岁、肿瘤＜1.5 cm、肿瘤位于肺下叶、单发肿瘤穿刺肺组织次数＞3次、消融多个肿瘤穿刺次数多、消融路径穿过肺组织的长度较长或者穿过较大的叶间裂。大部分气胸容易治疗，或者是自限性的，不需要治疗即可自愈，需要胸腔闭式引流的患者占3.5%-40%^[19, 117, 119, 120]^。如果患者经过胸腔闭式引流仍然有气体漏出，可以持续负压吸引、行胸膜固定术、气管镜下注入硬化剂、气管内置入阀门等^[123]^。另外，要注意迟发性气胸的发生，一般认为消融后72 h后发生的气胸称为迟发性气胸^[124]^。

#### 11.2.2 胸腔积液

消融后经常可以见到少量胸腔积液，发生率为1%-60%^[119, 122]^，被认为是机体对热损伤的交感反应，需要穿刺/置管引流的胸腔积液占1%-7%。导致胸腔积液发生的危险因素有：大病灶、一次消融多个病灶、病灶靠近胸膜（< 10 mm）、消融时间长等^[125]^。

#### 11.2.3 出血

消融中出血的发生率在3%-8%^[119, 126, 127]^，出血主要表现为咯血、血胸、失血性休克和急性呼吸衰竭，但主要表现为咯血和血胸。①咯血：在消融过程中大咯血的发生率很低。肺内出血导致咯血常见于以下情况^[126, 127]^：a.病灶直径＜1.5 cm，小病灶多需要更多地调整进针来进入靶点；b.中下肺野的病灶，此处的病灶更容易受到呼吸动度的影响，较难穿刺，并且针尖的运动更易损伤血管；c.穿过肺组织的针道长度超过2.5 cm，这类病灶更靠近肺门，周围大血管多，并且消融中需要损伤更多的肺组织；d.消融路径穿过肺血管，避免穿过血管可以避免多达80%的肺出血，平行而不是垂直于血管进针可以最大限度地避免此危险因素；e.应用多极消融针。如果出现中等以上的咯血时应立即消融，同时静脉输注止血药。由于消融本身可以使血液凝固，随着消融治疗的进行出血会逐渐停止，故在具体消融治疗过程中大出血的发生率并不高。在穿刺过程中应尽量避免穿刺到较大血管或者不张的肺组织等。术后咯血，多具有自限性，可持续3 d-5 d。保守治疗无效者，可行介入栓塞治疗或剖胸探查；②血胸：主要是在穿刺过程中损伤了胸廓内动脉、肋间动脉或其他动脉等。在穿刺过程中要避免穿刺到上述动脉，如果出现血胸要密切观察积极保守治疗，保守治疗无效者，可行介入栓塞治疗或剖胸探查。

#### 11.2.4 感染

消融手术引起的肺部感染的发生率为1%-6%^[117-119, 122, 126, 128]^，但是肺部肿瘤特别是NSCLC行消融治疗时患者多是无法耐受手术治疗的老年患者，常伴有基础的肺部疾患，肺部的感染和炎症会导致肺功能的急剧下降，甚至导致患者死亡。术前30 min-1 h可以预防性应用抗生素，24 h内再用一次。在下列情况下消融手术后预防性应用抗生素可以适当延长到48 h-72 h：老年人＞70岁、长期慢性阻塞性肺气肿、糖尿病控制欠佳、肿瘤＞4 cm、单侧肺肿瘤数量＞3个、免疫力低下等。若消融手术后5 d体温仍然＞38.5 ℃，首先要考虑肺部感染，要根据痰液、血液或脓液培养的结果调整抗生素。如果发生肺部或胸腔脓肿可以置管引流并冲洗。另外，接受过胸部放疗的患者易发生间质性肺炎，在此基础上行消融术者更易继发感染，要引起注意。

#### 11.2.5 空洞形成

空洞形成是肺部肿瘤热消融后的常见征象，可以视为术后的自然转归过程，但是也可能成为感染、出血等严重并发症的根源。空洞形成的发生率约14%-17%^[122, 129, 130]^，大多术后1个月-2个月出现，2个月-4个月后吸收。肿瘤临近胸壁、复发肿瘤和合并肺气肿的肿瘤，更易于出现空洞形成。大部分空洞没有症状，仅需观察不需处理。如果出现发热、衰弱，应考虑空洞感染、脓肿形成。另外，要警惕曲霉菌感染^[131-133]^。空洞引起的反复出血如果保守治疗效果不佳时可以用介入栓塞治疗。

#### 11.2.6 其他少见并发症^[134-140]^

支气管胸膜瘘、急性呼吸窘迫综合症、非靶区热灼伤或冻伤、肋骨骨折、冷休克、血小板降低、肿瘤针道种植、神经损伤（臂丛、肋间、膈、喉返等神经）、肺栓塞、空气栓塞、心包填塞等均有个案报道，需个别特殊处理。

#### 11.2.7 消融相关死亡

肺部肿瘤消融手术的并发症大多轻微且易于处理，但是严重甚至致命的并发症也有一定的发生率。根据目前的文献报导肺部肿瘤消融手术相关死亡率最低为0%^[118]^，最高2.6%^[141]^。美国报道了一组3, 344例肺部肿瘤消融手术的住院相关死亡率为1.3%。主要死亡原因为：各种肺炎（包括霉菌性肺炎）、肺脓肿、大出血/大咯血（包括肺动脉假性动脉瘤破裂出血）、支气管胸膜瘘、空气栓塞和急性呼吸窘迫综合征^[117]^。

## 12 消融和其他治疗联合

消融与其他方法进行联合治疗是目前许多肿瘤研究的重要内容之一，包括消融与外科、化疗、放疗和分子靶向药物等的联合。消融与放疗可以提高肿瘤的局部控制率，延长患者的生存期，而副反应无明显增加^[142-144]^。对于进展期NSCLC消融与化疗结合的研究逐渐增多，消融联合化疗对于提高肿瘤的局部控制率、延长患者的生存期有一定益处^[145-151]^，有可能成为治疗进展期NSCLC的新模式。酪氨酸激酶抑制剂（tyrosine kinase inhibitor, TKI）药物是目前治疗有表皮生长因子受体（epidermal growth factor receptor, EGFR）突变的进展期NSCLC的主要方法之一，这类患者应用TKIs可以获得约70%的客观缓解率及约10个月的无进展生存时间。然而在接受一段时间的TKI治疗后，几乎所有患者都会出现耐药。对于局部肿瘤缓慢进展和孤立性病灶进展的患者进行局部热消融治疗后，继续服用TKIs药物，可延长患者的中位无进展生存时间和总生存时间^[152-155]^。

## 13 结语

关于肺部肿瘤的治疗，微创治疗是未来发展的方向之一，尤其是影像引导下的经皮热消融技术在治疗肺部肿瘤方面具有：创伤小、疗效明确、安全性高、患者恢复快、操作相对简单、适应人群广等特点。最近研究^[156]^表明：经皮热消融治疗不能耐受手术切除早期NSCLC患者（肿瘤直径2 cm-3 cm）的1年、3年和5年的生存率分别达到97.7%、72.9%和55.7%，且死亡率小于1%。这些临床证据让我们相信未来这一技术会在肺部肿瘤的综合治疗中得到越来越广泛的应用，其地位有可能成为继手术、放疗、化疗之后的一种新的治疗模式。但是从临床实践的角度看，有关热消融技术治疗原发性和转移性肺部恶性肿瘤患者的例数与手术、放疗和化疗相比相对较少^[27, 157]^，需要进一步开展工作以改变传统肿瘤工作者对热消融技术的认知，使得该治疗方法得以普及和规范化应用。

目前热消融技术治疗原发性和转移性肺部恶性肿瘤还存在许多问题：①热消融技术已经成为肺部肿瘤多学科综合治疗领域的重要手段，特别是对于早期不能耐受外科手术切除的周围型肺癌患者有可能成为首选，但是尚缺乏大规模的、多中心的、随机的、前瞻性的临床研究；②缺乏与其他传统治疗手段（如立体定向放射治疗）的前瞻性的、多中心的临床比较研究；③热消融与其他治疗手段（如放疗、化疗和分子靶向药物治疗等）联合应用的临床研究相对较少；④如何提高局部完全消融率，降低局部复发^[158]^，也是今后工作的方向之一；⑤作为我国的“限制性医疗技术”，由于治疗设备的生产厂家不同，设备性能之间的差异，再加上该专业刚刚兴起，治疗人员的专业化水平参差不齐，现在很难形成公认的治疗规范；⑥应用热消融技术治疗后的疗效判断有时较难与现行的国际标准接轨，使用现有的影像学手段有时较难真实反映出热消融技术治疗后的疗效，因此，制定公认的、符合热消融技术自身规律的疗效判断标准还需要进行艰苦的工作；⑦姑息消融在肺癌综合治疗的位置还有待于进一步探讨；⑧基础研究相对滞后，如复杂热场分布、对机体免疫的影响等等。

## 附件 CT引导经皮穿刺肺部肿瘤热消融操作规程



（柳晨 林征宇 **执笔**）

对于已经具备经皮穿刺热消融治疗肺部肿瘤适应证的患者，规范化实施操作的具体步骤如下：

1、以术前CT扫描图像评估，将患者以合适的体位（俯卧位、侧卧位、仰卧位等）置于CT扫描床上，以患者舒适和稳定为宜，必要时采用束缚带或真空负压垫固定。

术前患者进行呼吸训练，建议采用平静呼吸状态下屏气。

2、将CT定位坐标尺纵向粘附在病灶所在区域的体表投影处，CT扫描（建议3 mm-5 mm层厚扫描）。

初步制定穿刺计划：

①确定病灶的位置、大小、形态、与邻近器官的关系；

②穿刺点的体表定位：经皮穿刺通过预设路径到达病灶的皮肤穿刺进针点（实际操作时为CT定位纵向与横向坐标尺交叉处）；

③选择路径：指从皮肤穿刺点到达病灶穿刺通道，此距离称为“靶皮距”。路径需满足穿刺点到达病灶有适当的距离（靶皮距>2 cm），病灶与邻近器官清晰可辨，能穿刺到病灶的最大截面，无骨骼、大血管、气管或其它重要组织结构阻挡；

④分别测量进针角度以及皮肤穿刺点距离壁层胸膜和病灶的距离，必要时还需测量穿刺路径上距重要组织结构的距离；

⑤一般选取较大肋间隙进行操作，便于适当调整穿刺方向。必要时可采用消融穿刺的辅助技术如：人工液胸或人工气胸。

3、以1%-2%利多卡因局部逐层浸润麻醉，必要时行胸膜麻醉。麻醉满意后，可以将注射器针头留置于体表，行CT扫描，以其为标记初步观察、模拟消融穿刺进针角度。

4、尖刀片在进针点处破皮，在CT扫描监视下，将消融针按预设的穿刺路径逐步穿刺到达靶病灶。

建议采用三步法：

①对于胸壁较厚者在消融针穿刺至壁层胸膜未进入肺组织前或对于胸壁较薄者在消融针穿刺入少许肺组织后，行CT扫描观察进针角度及穿刺路径上的重要组织结构；

②消融针穿刺接近靶病灶时，行CT扫描观察：进针角度、与邻近重要组织结构的关系及穿刺路径上是否有出血或气胸等并发症发生；

③消融针穿刺入靶病灶后，行CT扫描（必要时可行三维重建）确认消融针在靶病灶内的位置及与周围重要组织结构的关系。在穿刺消融过程中如出现大量咯血或大量气胸应及时处理。

5、根据不同设备所使用的消融参数（温度、功率、时间、循环等）进行消融治疗，在消融过程中应用CT扫描监测消融针是否脱靶、是否需要调整消融针的深度和角度、是否达到了预定消融范围、是否出现术中并发症（如出血、气胸等）。

6、消融结束后，行针道消融并缓慢拔出消融针。针道消融要避免损伤胸膜及皮肤。

7、术后全肺CT扫描观察：是否有即刻并发症及初步判断疗效。在消融后如出现大量胸腔积血或积液、大量气胸等并发症应及时处理。
